# Clinical benefits of novel non‐nucleoside reverse transcriptase inhibitors: A prospective cohort study

**DOI:** 10.1002/iid3.1217

**Published:** 2024-04-05

**Authors:** Shujing Ma, Xiaoxin Xie, Yanhua Fu, Lin Gan, Xiaoyan Yang, Linghong Kong, Jun Li, Hai Long

**Affiliations:** ^1^ School of Public Health, the Key Laboratory of Environmental Pollution Monitoring and Disease Control, Ministry of Education Guizhou Medical University Guiyang Guizhou China; ^2^ Department of Infection Guiyang Public Health Clinical Center Guiyang Guizhou China

**Keywords:** Effectiveness and safety, Non‐nucleoside reverse transcriptase inhibitors, HIV symptom index, Patient‐reported outcomes, Pittsburgh Sleep Quality Index

## Abstract

**Introduction:**

The efficacy and safety of ainuovirine+lamivudine+tenofovir (ANV+3TC+TDF) and efavirenz+lamivudine+tenofovir (EFV+3TC+TDF) have been confirmed in previous clinical trials; however, there are no related studies on patient‐reported outcomes. This study aimed to evaluate the effectiveness and safety of these 2 antiretroviral therapy regimens and to understand the patient's symptom experience and subjective experience of sleep quality through patient‐reported outcomes.

**Methods:**

This is a single‐center prospective cohort study with 243 patients evaluated from October 1, 2021 to June 30, 2022. Virological effectiveness and patient‐reported outcomes results were analyzed. The primary endpoint was the proportion of HIV viral load <50 copies/mL (virological suppression rate) at 48 weeks and the changes in the HIV symptom index and Pittsburgh sleep quality index.

**Results:**

The virological suppression rates in the ANV+3TC+TDF and EFV+3TC+TDF groups were 83.6% (102/122) and 87.6% (106/121), respectively, at 48 weeks. In the ANV+3TC+TDF group, the scores of HIV symptom index and pittsburgh sleep quality index in the 48th week were lower than the baseline level (*p* < 0.05). Logistic regression results showed that the baseline regimen EFV+3TC+TDF was a risk factor for dizziness/lightheadedness (odds ratio = 3.153, 95% confidence interval: 1.473–6.748, *p* = 0.003), sadness/depression odds ratio = 2.404, 95% confidence interval:1.188–4.871, *p* = 0.015), and difficulty sleeping (odds ratio = 2.802, 95% confidence interval: 1.437–5.463, *p* = 0.002) at 48 weeks.

**Conclusions:**

Both regimens showed good virological effectiveness; however, compared with ANV+3TC+TDF, the EFV+3TC+TDF regimen reduced the prevalence of HIV‐related symptoms.

## Graphical abstract

Both regimens showed good virological efficacy; however, compared with EFV/3TC/TDF, the ANV/3TC/TDF regimen reduced the prevalence of HIV‐related symptoms.

## 1 INTRODUCTION

Antiretroviral therapy(ART) is the most effective method to treat human immunodeficiency virus/acquired immune deficiency syndrome (HIV/AIDS) at present, which can significantly reduce mortality and prolong the life of patients.^1^ However, prolonging life does not necessarily improve the quality of life (QoL). During the long‐term ART treatment, people living with HIV (PLWH) will face many physical and mental health problems, including pain, insomnia, fatigue and depression, which will seriously affect their quality of life and then affect their adherence to ART.^2^ Studies have shown that if patients experience a better quality of life, the spread of the disease to others is limited.^3^ Therefore, choosing the right ART regimen is important. The ART regimen recommended by most international guidelines for treating newly diagnosed patients with HIV/AIDS includes two nucleoside reverse transcriptase inhibitors (NRTIs) combined with a third drug,^1^, ^4^, ^5^ which may be non‐nucleoside reverse transcriptase inhibitors (NNRTIs), protease inhibitors (PIs), or integrase transfer inhibitors (INSTIs). Currently, many low‐ and middle‐income countries are still dominated by NNRTIs, partly due to limited drug accessibility.^6^ In China, EFV+3TC+TDF is the primary regimen, which is used by over 80% of PLWH.^7^, ^8^ However, many clinical trials and cohort studies have reported that adverse reaction symptoms of EFV, such as central nervous system disorders, rash, and dyslipidemia seriously affected the quality of life of patients, led to worse adherence, and finally failed in treatment.^9^, ^10^ Ainuovirine (ANV)^11^ is a newly developed NNRTI in China; in 2021, it was recommended as a first‐line regimen by China AIDS diagnosis and treatment guidelines, and it became a new choice for the treatment of PLWH. Preclinical and phase 1 clinical studies have shown that ANV has low drug interaction potential, good pharmacokinetics/pharmacodynamic characteristics, and good tolerance without serious adverse reactions.^12^, ^13^ Previous studies have shown that the efficacy of ANV+3TC+TDF and EFV+3TC+TDF in clinical trials can reach more than 90%,^11^ however, the tolerance and acceptance of long‐term medication will affect the compliance and persistence of this therapy, so it is important to prove the comparative influence of these two schemes on QoL measures.

Physiological indicators such as CD4+T cell number and viral load do not reflect the subjective feelings of patients with AIDS^14^; therefore, they cannot be used to evaluate the health QoL of patients. Currently, one of the most effective methods for evaluating health QoL is the collection and analysis of patient‐related outcomes (PROs), which measure patients' health status and are reported directly by patients without the need for doctor explanations.^15^ The evaluation and application of PRO data are increasingly being applied in clinical trials; however, in the clinical treatment of AIDS in China, the application of PRO is still in the initial exploration stage. The HIV symptom index (HIV‐SI) and Pittsburgh Sleep Quality Index (PSQI) are PRO instruments widely used to evaluate HIV‐related symptoms and sleep quality.^16^

Therefore, this study aimed to better understand the experiences of PLWH using ANV+3TC+TDF and EFV+3TC+TDF. We used the two previously verified PRO scales to evaluate the experience and sleep quality of patients with common symptoms related to HIV infection or treatment after undergoing two ART regimens. Our purpose was to collect patients' experiences and subjective feelings through PRO while evaluating the effectiveness and safety of these two ART regimens to obtain a more comprehensive view of their health status.

## 2 METHODS

### 2.1 Study design and participants

This single‐center prospective cohort study was conducted at the Guiyang Public Health Clinical Centre (one of China's largest infectious disease hospitals). Patients were enrolled from October 1, 2021, to June 30, 2022 and followed up until August 2023. Inclusion criteria: 1) adults (≥18 years old) with HIV‐1 antibody confirmed positive; 2) never previously received antiviral treatment (ART‐naïve); 3) ART regime was ANV+3TC+TDF or EFV+3TC+TDF; Exclusion criteria: 1) pregnant women; 2) patients with severe hepatic and renal insufficiency; and 3) patients who failed to complete the HIV‐SI and PSQI questionnaires at the time of the interview.

### 2.2 Study endpoints

Main endpoints and indicators: (1)The ratio of HIV viral load <50 copies/mL at 48 weeks (virological suppression rate); (2)The changes in HIV‐SI and PSQI scores compared with the baseline at the 48th week. The secondary endpoints included: (1) Changes in CD4 and CD4/CD8 in the ANV+3TC+TDF and EFV+3TC+TDF groups at week 48; (2) changes in blood biochemistry, blood lipids, weight and body mass index (BMI) at the end of week 48.

### 2.3 Definitions

Virological suppression (VS): plasma HIV‐RNA viral load (pVL) < 50 copies/mL^1^; Baseline: The first day of ART; BMI: >29.9 kg/m2 is obesity; 25 ~ 29.9 kg/m^2^ is overweight; 18.5 ~ 24.9 kg/m^2^ normal; less than 18.5 kg/m^2^ is thin.^17^ dyslipidemia was defined as high total cholesterol (TC ≥ 5.2 mmol/L), high triglyceride (TG ≥ 1.7 mmol/L), low high‐density lipoprotein cholesterol (HDL‐C < 1.0 mmol/L), or high low‐density lipoprotein cholesterol (LDL‐C ≥ 3.4 mmol/L). Dyslipidemia was present if any of these four lipid parameters were abnormal.^18^ Blood routine test is the most basic examination. Blood routine test is the most basic examination. The blood routine test results include red blood cell count (RBC), hemoglobin (HGB), platelets (PLT), white blood cell count (WBC), lymphocyte count (LYMC), lymphocyte ratio (LYMPH), neutrophil count (NEUT), neutrophil ratio (NEU) neutrophil to lymphocyte ratio (NLR) The urine routine test is a combination of tests that are performed on urine and is one of the most frequently conducted tests. It comprises the physical, chemical and microscopic examination of urine.

### 2.4 Procedures

Follow‐up assessments were conducted from the day of ART initiation. Patients received either the ANV+3TC+TDF or EFV+3TC+TDF regimen, and the day ART was initiated was recorded as the baseline date. Virus load, CD4 count, blood routine, urine routine, blood biochemistry, hepatitis B virus (HBV), hepatitis C antibody, drug resistance test, electrocardiogram, chest X‐ray, ultrasound, and other related tests were performed at the baseline visit follow‐up was conducted at 4, 12, 24, 36, and 48 weeks. Participants were advised to have a blood routine, blood biochemical and urine tests at each visit, and additional CD4 and viral load tests at 8, 24, and 48 weeks, although these tests were not mandatory.

### 2.5 Patient‐reported outcome measure and instrument administration

To better understand the incidence of HIV symptoms and the changes in sleep quality after adopting the two regimens, we measured the patients with the PRO scale. Study participants need to fill out questionnaires at baseline and at the 48th week of follow‐up. If the participants filled out HIV‐SI and PSQI questionnaires at baseline and at week 48, the PRO data of the participants were included in this study. Participants will be excluded if they miss one item in the questionnaire at baseline or at week 48. Participants with illiteracy, presbyopia or dyslexia were interviewed by their AIDS case managers.

The HIV‐SI is a validated PRO scale with a Cronbach'α scale of 0.79 and the test‐retest correlation coefficient is 0.92. It is used to evaluate the burden of 20 common symptoms related to HIV treatment or disease^19^ and is considered the gold standard for studying contemporary HIV symptoms.^20^ Respondents were asked about their experience with each of the 20 symptoms in the past 4 weeks, using a 5‐point Likert scale. The answer options and scores are as follows: (0) “I don't have this symptom”; (1) “I have this symptom, and it doesn't bother me”; (2) “I have this symptom and it bothers me a little”; (3) “I have this symptom and it bothers me”; (4) “I have this symptom and it bothers me a lot.”

The PSQI is a validated 19‐item scale to evaluate sleep quality and interference within 1 month. The Cronbach'α of the scale is 0.796. Its scoring items are divided into seven dimensions of quality factor scores: “time to fall asleep, sleep efficiency, sleeping drugs, sleep time, sleep quality, sleep disorder, and daytime dysfunction.” Each quality factor is scored according to a score of 0–3, and the scores of each quality factor are accumulated to form the total factor of PSQI. The total score of PSQI ranges from 0 to 21. The lower the score, the better the sleep quality, and PSQI ≥ 5 is classified as a sleep disorder.^21^

### 2.6 Statistical analysis

Excel 2016 (Microsoft, Redmond, Washington, USA) was used to input the data, and SPSS (version 23.0; IBM, Armonk, NY, USA) was used for statistical analysis. Data were expressed as mean ± standard deviation or median (interquartile range [IQR]) according to the data distribution type. The Kolmogorov–Smirnov test was used to determine whether numerical variables fit the assumptions for normality of distribution. Classification data were compared using chi‐square or Fisher's exact tests. Depending on the distribution, a t‐test or Wilcoxon rank‐sum test was used to analyze continuous data. All statistical tests were bilateral; a *p* value < 0.05 was considered statistically significant. To better understand the influence of HIV symptoms in the two regimens, univariate and multivariate logistic regression models were used to evaluate the influence of the parameters (age, sex, HBV, DM, cardiovascular disease, baseline CD4 T cell count, 48‐week viral load <50 copies/mL, and baseline ART regimen on the symptoms). The results of the logical model consisted of the odds ratio (*OR*), 95% confidence interval (95% *CI*), and *P* value of the Wald test.

## 3 RESULTS

### 3.1 Patient characteristics

**Figure 1:**
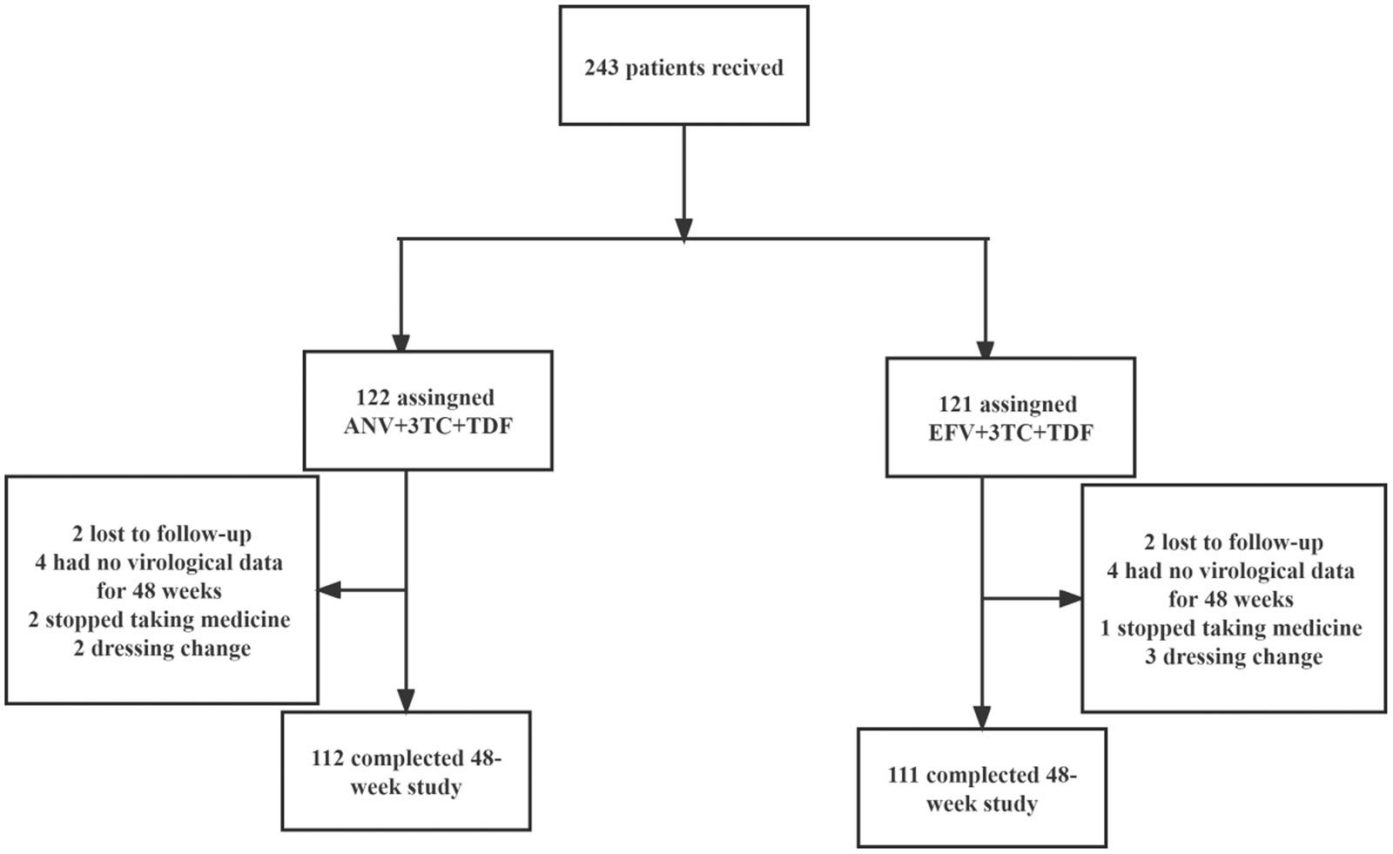
Patient flow chart.

The patient selection process is illustrated in Figure 1. Between October, 1, 2021 and June 30, 2022. 243 ART‐naïve patients were assessed. All participants received the ANV+3TC+TDF or EFV+3TC+TDF regimen. Among these patients, 122 received ANV+3TC+TDF, of which two participants were lost to follow‐up, two changed their ART regimen, two stopped ART, and four had no data at 48 weeks; therefore, 112 patients completed 48 weeks of follow‐up. In total. 121 participants received EFV+3TC+TDF, of whom two were lost to follow‐up, three changed their ART regimen, one stopped ART, and four had no data at 48 weeks; therefore, 111 patients completed 48 weeks of follow‐up (Figure 1).

In the ANV+3TC+TDF group, 78 patients (63.9%) were male, with a median age of 41.0 (30.8–56.0) years, and 90 patients (73.8%) had heterosexual transmission. There were 88 patients (72.1%) with a CD4 cell count of ≥200 cells/μL at baseline. The baseline viral load of 28 patients (23.0%) was ≥1 × 10^5^ copies/mL. Among the 122 patients, three were diabetic, two had HBV, seven had cerebrovascular disease, and 21 had opportunistic infections (Table 1).

**Table 1:** Baseline characteristics of two groups of patients.

Characteristic	ANV+3TC+TDF (N = 122)	EFV+3TC+TDF (N = 121)	*P* value
Sex, n(%)			0.141
Male	78 (63.9)	88 (72.7)	
Female	44 (36.1)	33 (27.3)	
Median age (IQR), year	41.0 (30.8‐56.0)	44.0 (30.0‐56.5)	0.242
Transmission routen (%)			0.322
Homosexual transmission	32 (26.2)	28 (23.1)	
Heterosexual communication	90 (73.8)	91 (75.2)	
Injecting drugs	0 (0.0)	2 (1.7)	
Median CD4 count (IQR), cells/μL	322.5 (140.8‐411.3)	306.0 (212.0‐442.0)	0.412
<200 cells/μL, n(%)	39 (32.0)	28 (23.1)	0.124
≥200 cells/μL, n(%)	83 (68.0)	93 (76.9)	
HIV viral load, copy/mL, n(%)			0.209
<100000	94 (77.0)	101 (83.5)	
≥100,000	28 (23.0)	20 (16.5)	
BMI, mean ± SD, kg/m^2^	21.9 ± 3.3	21.8 ± 2.8	0.073
Complicated disease, n(%)			
DM	3 (2.5)	2 (1.8)	1.000
Cardiovascular disease	7 (5.7)	5 (4.5)	0.769
HBV	2 (1.8)	7 (6.3)	0.087
AIDS‐related opportunistic infections, n(%)	21 (17.2)	17 (15.3)	0.497

Abbreviation: AIDS, acquired immunodeficiency syndrome; Plasma HIV‐RNA, viral load; ANV+3TC+TDF, Ainuovirine+lamivudine+Tenofovir; EFV+3TC+TDF, Efavirenz+lamivudine+Tenofovir; BMI, body mass index; HBV, hepatitis B virus; DM, diabetes mellitus; IQR, quartile distance.

In the EFV+3TC+TDF group, there were 88 males (72.7%) with a median age of 44.0 (30.0–56.5) years; 91 patients (75.2%) had heterosexual transmission, and two cases (1.7%) were transmission via drug injection. There were 93 patients (76.9%) with a CD4 cell count of ≥200 cells/μL at baseline. The baseline viral load of 20 patients (16.5%) was ≥1 × 10^5^ copies/mL. Among the 121 patients, two were diabetic, seven had HBV, five had cerebrovascular disease, and 17 had opportunistic infections (Table 1).

### 3.2 Virus effectiveness

**Figure 2:**
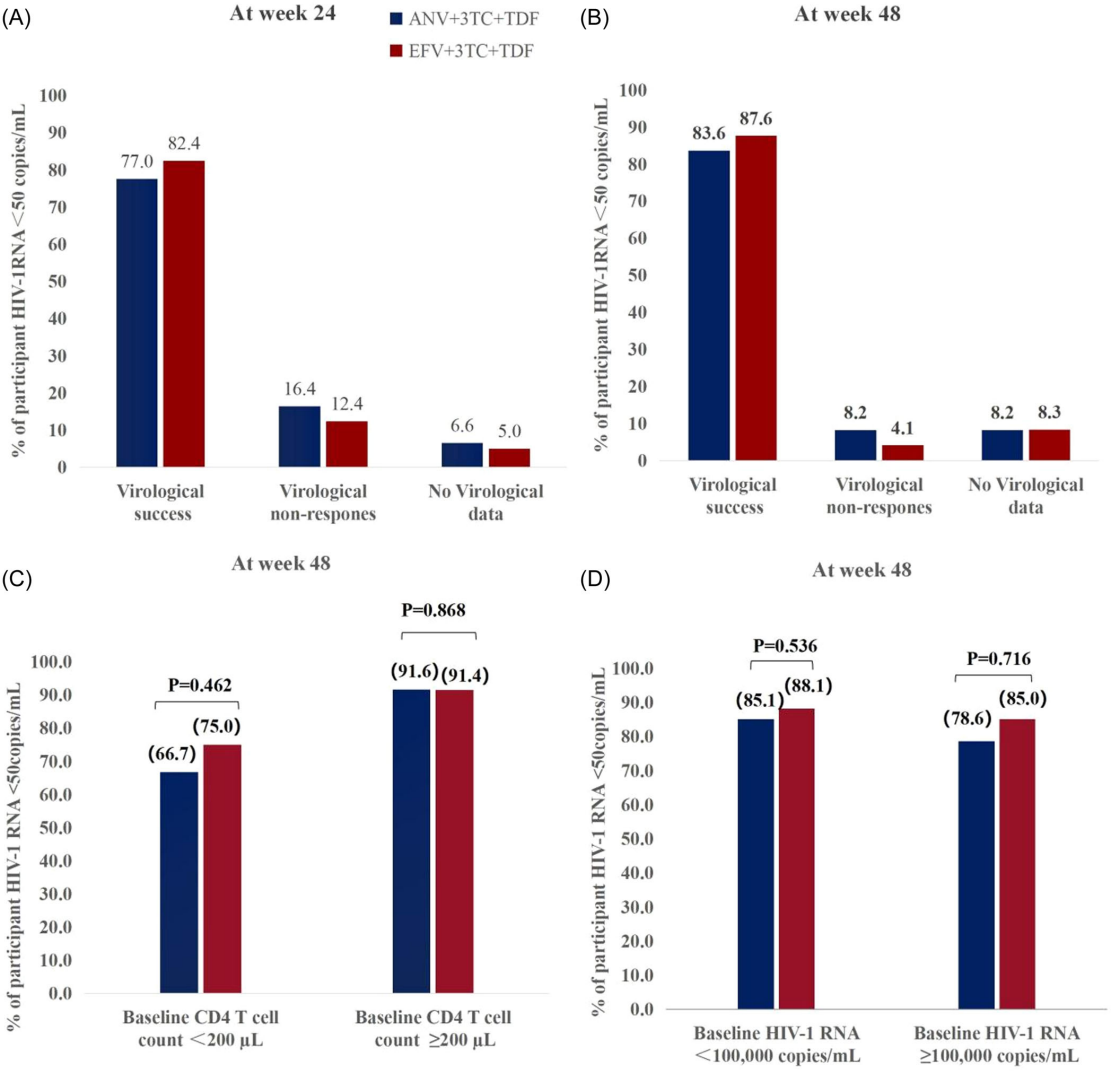
Snapshot analysis of the proportion of HIV‐1 RNA <50 copies/mL participants. (A) the proportion of participants with HIV‐1 RNA <50 copies/mL in the 24th week in the two groups; (B) the proportion of participants with HIV‐1 RNA <50 copies/mL in the 48th week in the two groups; (C) percentage of participants with HIV‐1 RNA <50 copies/mL divided into baseline CD4 T cell count of <200 cells/μL and ≥200 cells/μL at week 48; (D) Percentage of participants with HIV‐RNA <50copies/mL divided into baseline concentrations of <100,000 copies/mL and ≥100,000 copies/mL at 48 weeks. Abbreviations: ANV, Ainuovirine; EFV, Efavirenz.

Figure 2A shows the virological results of two groups of patients at the 24th week. The virological suppression rate of patients in the ANV+3TC+TDF group was 77.0% (94/122), and that of patients in the EFV+3TC+TDF group was 82.4% (100/121). In the ANV+3TC+TDF and EFV+3TC+TDF groups, the proportion of patients without virological data at 24 weeks was 6.6% (8/122) and 5.0% (6/121), respectively.

Figure 2B shows the virological results of two groups of patients at the 48th week. At week 48, the virological suppression rate of the ANV+3TC+TDF group was 83.6% (102/122), and that of the EFV+3TC+TDF group was 87.6% (106/121). In the ANV+3TC+TDF and EFV+3TC+TDF groups, the proportion of patients who failed virology at 48 weeks was 8.2% (10/122) and 4.1% (5/121), respectively.

In a dedicated sub‐analysis, we found no difference in the proportion of VS in patients with CD4 < 200 cells/μL between the ANV+3TC+TDF and EFV+3TC+TDF groups (66.7% vs. 75%, respectively; *p* = 0.462). In the subgroup of patients with CD4 ≥ 200 cells/μL, the proportion of VS in the ANV+3TC+TDF and EFV+3TC+TDF groups was 91.6% and 91.4%, respectively (*p* = 0.868)(Figure 2C). Stratified analysis of baseline HIV‐1 RNA showed that the VS rates of patients with baseline HIV‐1 RNA < 1 × 10^5^ copies/mL were 85.1% and 88.1% in the ANV+3TC+TDF and EFV+3TC+TDF groups, respectively (*p* = 0.536). In patients with HIV‐1RNA ≥ 1×10^5^ copies/mL, the VS rates in the ANV+3TC+TDF and EFV+3TC+TDF groups were 78.6% and 85.0%, respectively (*p* = 0.716) (Figure 2D).

### 3.3 Immunological effectiveness

Table 2 shows the immunological changes of the two groups after 48 weeks of treatment. After the 48‐week follow‐up, the mean change in the CD4+ lymphocyte count increased by 94.5 (44.0, 175.0) cells/μL in the ANV+3TC+TDF group and 107.0 (56.0, 203.0) cells/μL in the EFV+3TC+TDF group (*p* = 0.407). In the ANV+3TC+TDF and EFV+3TC+TDF groups, the CD4/CD8 ratio increased by 0.24（0.13, 0.44）and 0.31 (0.12, 0.49), respectively (*p* = 0.225).

**Table 2:** Changes in CD4 cell counts and CD4/CD8 at week 48.

	ANV+3TC+TDF (n = 112)	EFV+3TC+TDF (n = 111)	*P*
CD4 cell count (cells/μL)			0.407
Baseline	324 (150.8, 411.8)	306.0 (212.0, 442.0)	
Mean change from baseline at week 48	94.5 (44.0, 175.0)	107.0 (56.0, 203.0)	
CD4/CD8			0.225
Baseline	0.32 (0.23, 0.42)	0.32 (0.21, 0.50)	
Mean change from baseline at week 48	0.24 (0.13, 0.44)	0.31 (0.12, 0.49)	

### 3.4 Changes of body weight, body mass index and biochemical indicators

**Figure 3:**
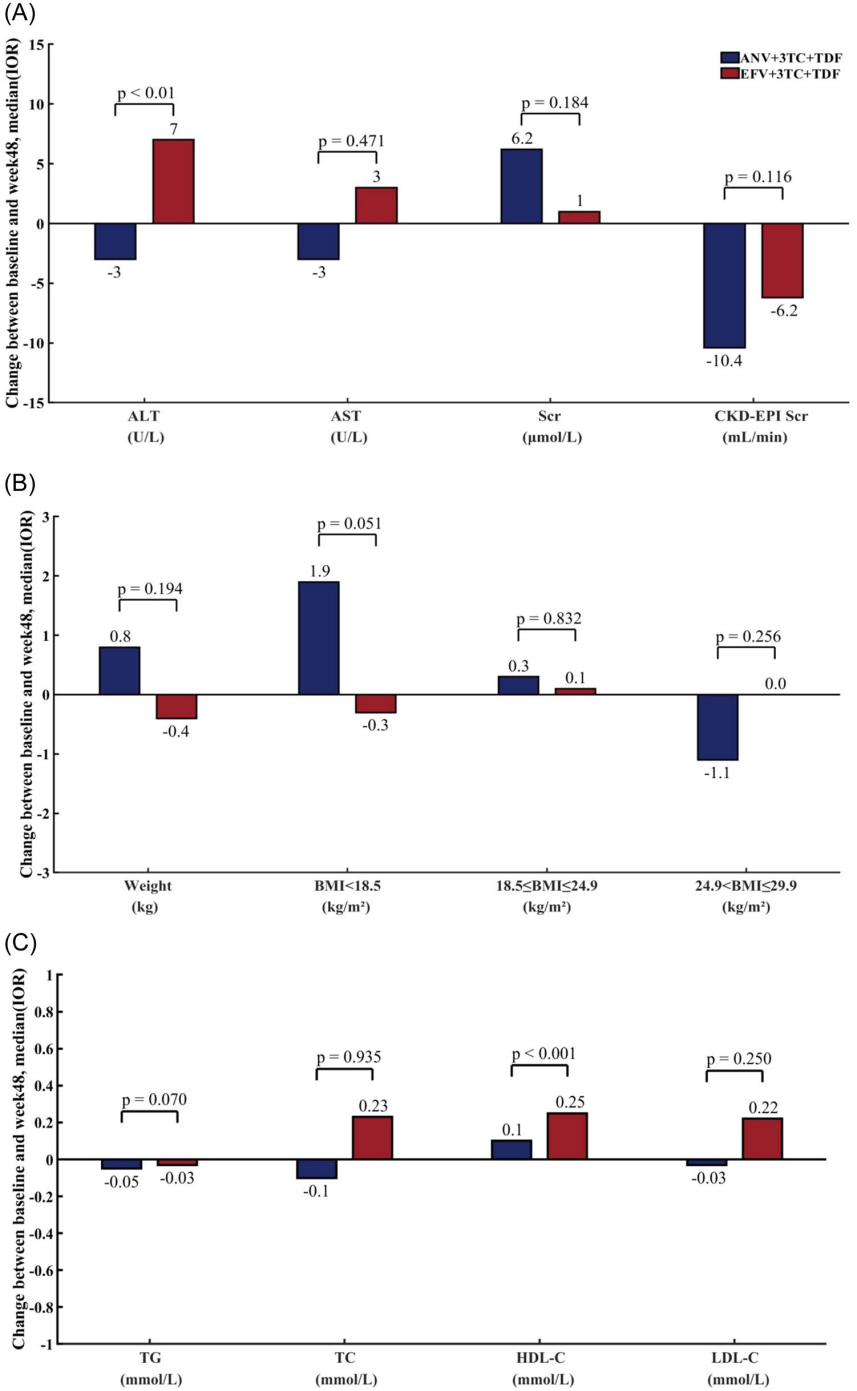
Changes of biochemical and immune indexes between baseline and 48 weeks. (A) Average changes in liver and kidney function indexes relative to baseline at the 48th week. (B) Average changes of body weight and body mass index from baseline at the 48th week. (C) Average changes of serum or plasma lipids from baseline at the 48th week. Abbreviations: ALT, alanine aminotransferase; AST, aspartate aminotransferase; BMI, body mass index changes; Scr, serum creatinine; CKD‐EPI Scr, Serum creatinine clearance rate; HDL‐C, high density lipoprotein cholesterol; LDL‐C, low density lipoprotein cholesterol; TC, total cholesterol; TG, triglyceride.

In terms of liver parameters, the results show that. Compared with the baseline, the liver parameters (ALT) in the ANV+3TC+TDF group decreased by 3 (U/L), and the liver parameters (ALT) in the EFV+3TC+TDF group increased by 7 (U/L), with statistical differences (*p* < 0.05). Regarding renal parameters, the creatinine of the ANV+3TC+TDF group increased by 6.2 μmol/L, and there was no statistical difference between the two groups (Figure 3A).

Regarding BMI, there is no significant difference between the ANV+3TC+TDF and EFV+3TC+TDF groups in the 48th week (Figure 3B). In terms of lipid metabolism, at the 48th week, the increase of high‐density lipoprotein was observed in both treatment groups, and the difference was statistically significant. The changes in other indexes were not statistically significant. (Figure 3C).

### 3.5 PRO results

After 48th weeks of using ANV+3TC+TDF and EFV+3TC+TDF, the scores of (HIV‐SI and PSQI of patients are summarized as follows. In the ANV+3TC+TDF group, compared with the baseline, at the 48th week, the median scores of HIV‐SI and PSQI decreased. (HIV‐SI was 6.0 [2.0–12.0] vs.3.0 [2.0–6.0], *p* < 0.001, PSQI was 5.0 (4.0–9.0) vs.4.0 (2.0–5.0) (*p* < 0.001). In the EFV+3TC+TDF group, compared with the baseline, the median score of HIV‐SI in the 48th week decreased (5.0 [2.0–11.0] vs.4.0 (1.0–7.0), *p* = 0.035). The median score of PSQI did not change significantly (5.5 [4.0–8.0] vs.5.0 [3.0–7.0], *p* = 0.518).

In the ANV+3TC+TDF group, compared with the baseline, the proportion of symptoms, such as (fatigue/loss of energy, fevers/chills/sweats, dizziness/lightheadedness, sadness/depression, nervous/anxious, difficulty sleeping, coughing/trouble breathing, bloating/pain/gas in the stomach, and problems with sex)decreased at the 48th week, and the differences were statistically significant (*p* < 0.05). In the EFV+3TC+TDF group, compared to baseline, the proportion of fatigue/loss of energy and fevers/chills/sweats symptoms decreased in the 48th week, and the differences were statistically significant. At the 48th week, compared between the two groups, the incidence of dizziness, sadness/depression, nervousness/anxiety, difficulty in sleeping and skin problems/rash/itching in the ANV+3TC+TDF group was lower than that in the EFV+3TC+TDF group, and the difference was statistically significant (all *p* < 0.05) ((Table 3).

**Table 3:** Frequency of disturbing HIV symptoms in the two groups (n,%).

	Baseline	Weeks 48	Baseline	Weeks 48	
Individuals reporting symptoms %	ANV+3TC+TDF (n = 112)	ANV+3TC+TDF (n = 112)	EFV+3TC+TDF (n = 111)	EFV+3TC+TDF (n = 111)	*P*
Fatigue/loss of energy	34.8*	22.3*	39.6**	27.0**	0.415
Fevers/chills/sweats	23.2*	9.8*	23.4**	12.6**	0.509
Dizzy/lightheadedness	21.4*	10.7*	25.2	22.5	**0.018**
Pain/numbness/tingling in hands/feet	19.6	17.9	16.2	13.5	0.373
Trouble remembering	20.5	16.1	17.1	19.8	0.466
Nausea/vomiting	11.6	8.0	15.3	12.6	0.261
Diarrhea/loose bowels	22.3	16.1	23.4	18.0	0.699
Sad/down/depressed	26.8*	15.2*	30.6	27.0	**0.030**
Nervous/anxious	26.8*	16.1*	34.2	27.0	**0.047**
Difficulty sleeping	32.1*	15.2*	29.7	31.5	**0.004**
Skin problems/rash/itching	30.4	19.6	30.6	31.5	**0.042**
Coughing/trouble breathing	14.3*	3.6*	5.4	2.7	0.710
Headaches	14.3	6.3	6.3	4.5	0.533
Loss of appetite	12.5	8.0	13.5	9.0	0.795
Bloating/pain/gas in stomach	16.1*	6.3*	18.9	9.9	0.325
Muscle aches/joint pain	10.7	7.1	19.8	12.6	0.171
Problems with sex	9.8*	2.7*	14.4	6.3	0.191
Changes in body composition/Weight gain	13.4	8.9	12.6	13.5	0.278
Weight loss/wasting	9.8	6.3	9.0	4.5	0.244
Hair loss/changes	12.5	7.1	13.5	9.9	0.459

* indicates in the ANV+3TC+TDF group, the difference between baseline and 48‐week symptom ratio is statistically significant.

** indicates in the EFV+3TC+TDF group, the difference between baseline and 48‐week symptom ratio is statistically significant.

n is the number of participants with response for at least one symptom

The p‐value in bold type indicates that there was a statistical difference in the proportion of symptoms between the EFV+3TC+TDF group and the ANV+3TC+TDF group at 48 weeks. Abbreviation: ANV, ainuovirine; EFV, efavirenz.

### 3.6 Predictors of symptoms

Logistic regression analysis was performed on the statistically significant symptoms of the two groups after 48 weeks of univariate analysis, and the factors affecting dizziness/lightheadedness, sadness/depression, nervousness/anxiousness, skin problems/rash/itching, and difficulty sleeping were analyzed. Other covariables included in the multivariate model are sex, age, baseline CD4 T cell count <200 cells/µL or ≥200 cells/µL, 48‐week viral load <50 copies/mL, DM, HBV, cardiovascular disease, and baseline regimen of ANV+3TC+TDF or EFV+3TC+TDF. The results showed that EFV+3TC+TDF in the baseline regimen was a risk factor for dizziness/lightheadedness, sadness/depression, and difficulty sleeping at 48 weeks (Table 4).

**Table 4:** Logistic regression analysis of influencing five symptoms.

Symptom	Associated Factors	*OR* (95% *CI*)	*P* value
Dizziness/lightheadedness	ART (EFV vs. ANV)	3.153 (1.474–6.748)	0.003
Sadness/depression	ART (EFV vs. ANV)	2.406 (1.188–4.871)	0.015
Nervousness/anxiousness	ART (EFV vs. ANV)	1.653 (0.858–3.184)	0.133
Difficulty sleeping	ART (EFV vs. ANV)	2.802 (1.437–5.463)	0.002
Skin problems/rash/itching	ART (EFV vs. ANV)	1.872 (0.995–3.524)	0.052

Abbreviations: ART, antiretroviral therapy, OR, was obtained from multivariate analysis; HBV, hepatitis B virus; ANV, Ainuovirine; EFV, Efavirenz. The multivariate logistics regression model was used to adjust the following variables: sex, age, baseline CD4 and pVL, HBV, and hypertension.

## 4 DISCUSSION

To the best of our knowledge, this is the first real‐world study in China to evaluate the subjective feelings of patients with HIV treated with ANV+3TC+TDF and EFV+3TC+TDF with PRO data as the main outcome, supplementing the evidence from clinical randomized trials.

It is well established that the effects of ART can be evaluated using HIV‐1 RNA viral load and CD4 cell counts, and drug resistance can be monitored.^22^ At 48 weeks, the VS rates in the ANV+3TC+TDF and EFV+3TC+TDF groups were 83.6% and 87.6%, respectively. Compared to the third phase of the study,^11^ our VS rate was low. We believe the possible cause is due to a high incidence of AIDS‐related opportunistic infections and poor patient compliance. Research has shown that patients with AIDS‐related opportunistic infections have a higher risk of virological failure.^23^ Additionally, more complications indicate more drug combinations, possibly leading to drug interactions. In the 48th week, among the patients whose baseline HIV‐1 RNA was ≥1×10^5^ copies/mL and CD4 cell count was <200 cells/µL, the virological inhibition rate of the two groups was low. This trend has also been observed in other studies, where the immune response rate of patients with a high baseline viral load and low baseline CD4 cell count was low.^24^, ^25^ The CD4/CD8 ratio is also considered a marker of immune aging and a predictor of all‐cause death, which can provide the same prognostic information as the CD4 cell count.^26^ In this study, the CD4 cell count and CD4/CD8 ratio of the patients in the two groups increased after 48 weeks of treatment, indicating that the immune response measured by the increase in CD4 cell count was similar between the two treatment groups.

In this study, the ALT of both groups was higher than the baseline at the 48th week; however, both fluctuated within the normal range. Regardless, we do not think this has clinically significant implications. In the plasma lipid level, the increase in LDL‐C level is the most important risk factor for atherosclerotic cardiovascular disease (ASCVD), and the decrease in LDL‐C level can significantly reduce the risk of ASCVD morbidity and mortality.^27^ The prevalence of dyslipidemia, which is characterized by high LDL‐C levels, in adults with HIV/AIDS in China has reached 75.6%.^28^ In this study, the LDL‐C level of the ANV+3TC+TDF group showed a slight downward trend, whereas that of the EFV+3TC+TDF group increased. This shows that the ANV+3TC+TDF regimen may have less influence on blood lipids than the EFV+3TC+TDF regimen; however, long‐term effects require longer follow‐up.

PRO tools can be used in clinical research to gain a deeper understanding of the symptoms reported by patients because they may downplay or minimize these symptoms in the standard screening of adverse drug events.^29^ This phenomenon may be attributed to patients not reporting symptoms they are accustomed to or believe are difficult to treat. Patients are more likely to report symptoms if prompted by the PRO questionnaire. In this study, we asked patients to make subjective evaluations of two scales through PROs, focusing on the burden of 20 symptoms related to HIV disease or treatment and the quality of sleep. The results showed that compared with EFV+3TC+TDF, ANV+3TC+TDF reduced the burden of disease symptoms after 48 weeks of treatment. The obvious improvements are dizziness/dizziness, sadness/depression/depression, nervousness/anxiety, difficulty in sleeping and skin problems/rashes/itching. Now, ANV is a new generation of non‐nucleoside in China. ANV+3TC+TDF can avoid the limitations of EFV, such as adverse central nervous system reactions and skin problems. ANV may be superior to EFV in improving the quality of life of patients with ANV regimens.

The advantage of this study is that we evaluated the efficiency and safety of patients choosing two NNRTI regimens based on their subjective experiences of disease symptom burden and sleep quality using the PRO questionnaire, which combined the HIV‐SI and PSQI scales.

However, this study had some limitations. First, this was a single‐center, small‐sample, non‐randomized controlled study, and the patients were generally young and middle‐aged, which may lead to limited generalizability of the results. Second, because HIV is a complex disease, the answers to the questionnaire are subjective, and the mental state of the participants may influence them at a specific time. Thirdly, we have not calculated the sample size, which may lead to insufficient statistical power.

## 5 CONCLUSIONS

In conclusion, the research shows that the protocols based on ANV or EFV have similar virological and clinical results, but the QoL results are inconsistent. As measured by the PROs, the ANV+3TC+TDF regimen reduced the disease symptom burden and sleep quality problems of patients compared to the EFV+3TC+TDF regimen. This shows that choosing ANV+3TC+TDF as the treatment regimen has potential benefits for patients. In middle‐ and low‐income countries, ANV+3TC+TDF is a new treatment option for adults with HIV‐1 infection. A larger multicenter study should be conducted covering patients of different age groups and populations to increase the generalizability of the findings.

## AUTHOR CONTRIBUTIONS

Shujing Ma, Xiaoxin Xie and Yanhua Fu contributed to the conception and design of the research. The methodology and analysis plan were constructed by Hai Long and Jun Li. Hai Long and Lin Gan were responsible for the study design and analysis plan and carried out the data monitoring; Xiaoyan Yang performed the statistical analysis. Xiaoxin Xie, Yanhua Fu, and Linghong Kong contributed to the interpretation of data. Shujing Ma wrote the original draft. All authors substantially contributed to the conception and design of the article and interpreting the relevant literature and were involved in writing the article or revised it for intellectual content. All authors agreed on the submission of the manuscript to the journal and reviewed and agreed on all versions of the article before submission, during revision, the final version accepted for publication, and significant changes introduced at the proofing stage. All authors had access to the study data and take responsibility for the integrity of the data and accuracy of the data analysis.

## CONFLICT OF INTEREST STATEMENT

The authors have no relevant affiliations or financial involvement with any organization or entity with a financial interest in or financial conflict with the subject matter or materials discussed in the manuscript. This includes employment, consultancies, honoraria, stock ownership or options, expert testimony, grants or patents received or pending, or royalties.

## ETHICS APPROVAL STATEMENT AND INFORMED CONSENT

The study was performed in accordance with the principles of the Declaration of Helsinki, and prior approval was obtained from the institutional review board of the research center. Written informed consent was obtained from all study participants, and all participants had their records used in Ethics Committee of the Guiyang Public Health Clinical Center (No. 202306).

## PATIENT CONSENT STATEMENT

Written informed consent was obtained from the patients before the start of the study.

## Data Availability

The datasets used or analysed during the current study available from the corresponding author on reasonable request.
